# American Medical Association

**Published:** 1881-07-20

**Authors:** 


### *AMERICAN MEDICAL ASSOCIATION.*

In our last we sketched briefly the more important points in the proceedings of the American Medical Association. The hospitalities and courteseys of the occasion, we had not space to detail. Our Senior, Dr. T. S. Powell, who was present as a delegate from Georgia, and who is a Virginian by birth, enjoyed greatly what he is pleased to style the real Old Virginia hospitalities of the occasion.

He visited his old friends and relations in various sections of the State, spending many weeks. His gifts as a speaker are always called into requisition when he visits his native State. A subscriber to our Journal, writing from Brunswick, thus facetiously alludes to him :

"About the 24th I found your co-editor, Dr. Powell, in this county, and took him to Lawrenceville, where it being court week, he found many acquaintances. His reception by old friends, and also by many of the younger generation, anxious to shake hands with a *big man*, excelled anything I ever witnessed. Our Virginia people are very appreciative; and always delight to honor Virginia's sons who merit it."

He refers also to an address which Dr. P. made at Hicksford to a large audience. The Doctor also made an extempo address at a College Commencement in Brunswick, which was highly spoken of by the papers of the place for its ability and eloquence. That the Doctor had a fat time is evident, having returned home with an additional 20 pounds weight to his already Aldermanic proportions.

